# Methodology for evaluation of complex school-based health promotion interventions

**DOI:** 10.1057/s41271-024-00510-4

**Published:** 2024-08-12

**Authors:** Yvonne O’Byrne, J. Dinneen, T. Coppinger

**Affiliations:** Sport, Leisure & Childhood Studies, Munster Technological University, Rossa Avenue, Bishopstown, County Cork T12 P928 Ireland

**Keywords:** School, Intervention, Health promotion, Process evaluation

## Abstract

There is a need for careful examination of large volumes of collected (structured and unstructured) information related to school-based evaluation. There is also no published, comprehensive framework/s for evaluating complex interventions in Irish primary schools. The aim of this paper is to outline a methodology for process evaluation of an Irish primary school-based physical activity (PA) and nutrition intervention. Evaluation followed the three themes outlined by the British Medical Research Council: implementation, context, and mechanism of impact that we further divided into six dimensions. Methodological tools included questionnaires, PA logs, reflective journals, write and draw, and semi-structured interviews. We triangulated findings across these multiple tools to assess each dimension. We designed a unique framework to enable comparisons and offer researchers a template for evaluating complex health promotion interventions in primary schools. We present a methodology for evaluating a complex school-based health promotion intervention. The framework we propose integrates process and outcome data. It aims to enhance future result interpretation and facilitate informed comparisons among intervention schools.

**Supplementary Information:**

The online version contains supplementary material available at 10.1057/s41271-024-00510-4.

## Key messages

- Irish primary schools lack a comprehensive framework for evaluating complex health promotion interventions. By combining process and outcome data, researchers can assess intervention effectiveness across different schools.
- This study introduces a unique framework that will support informed comparisons.

**Supplementary Information:**

The online version contains supplementary material available at 10.1057/s41271-024-00510-4.

## Introduction

Complex school-based health promotion interventions play a crucial role in promoting health among children and adolescents [1]. Their evaluation is important so that policymakers, educators, and health professionals can use the information gathered to make informed choices about implementing or scaling up programmes. Although these types of interventions are increasing [2–4], their evaluation remains a complex process that requires careful consideration of several factors [5]. Substantial barriers related to their implementation and evaluation may be pertinent as developing, coordinating, and sustaining the partnerships can be difficult when there is a limited history of people from various disciplines working together [6]. Furthermore, there is a need for careful examination of large volumes of collected (structured and unstructured) information related to school-based evaluation.

Project Spraoi was an Irish school-based intervention [7] inspired by the fully evaluated ‘Project Energize’ (PENZ) [8]. Project Spraoi aimed to enhance children’s health and well-being by promoting PA and healthy eating across the entire school system. Key to the Project Spraoi approach was the ‘Energiser;’ a PA specialist who led health-promoting initiatives and who were also postgraduate researchers, who evaluated the different components of the programme [9–17]. The Energiser facilitated structured PA sessions called ‘huff & puff,’ conducted healthy-eating workshops, and provided resources to help teachers achieve the daily goal of 20 min of moderate to vigorous PA (MVPA), in addition to regular physical education (PE) classes. On days when the Energiser was absent, class teachers took over, and parents were encouraged to support the project through after-school healthy-eating workshops and assisting with ‘active’ homework.

A review of school-based interventions highlighted a need to better understand how implementation of multi-component interventions deals with various intervention targets [18]. Yet, studies of this type do not always include a systematic evaluation of how the intervention was delivered or received in context, or both. This can lead to a lack of transparency and reproducibility of the study results [5]. Process evaluation fills this gap. Documenting characteristics of the intervention adds value to analysis of multicomponent school-based interventions. Process evaluation also provides researchers with information about barriers to, or facilitators of, the intervention, or both, and its components, in specific contexts [19]. Such data enable policy makers and implementers to fine tune intervention activities as they transfer intervention theory to practice, or to new contexts, and design new health promotion interventions targeting children in a similar environment. Yet, process evaluations in the literature are reported to be of mixed quality and lack theoretical guidance [20].

Multiple frameworks highlight important aspects for consideration in development of process evaluation methods for complex interventions [21–25]. Few frameworks provide a workable template for implementation of a comprehensive process evaluation methodology in primary schools, particularly in Ireland.

In building upon previous literature, the British Medical Research Council’s (MRC) 2015 [19] Guidance for Process Evaluation of Complex Interventions outlines how to conduct and report process evaluations of complex interventions. The guidance recognises the value of process evaluation, stating that it can be used to assess fidelity and quality of implementation, clarify causal mechanisms and identify contextual factors associated with variation in outcomes. The guidance also provides a systematic approach to designing and conducting process evaluations, drawing on clear descriptions of intervention theory, and identification of key process questions. This framework uses three key themes for process evaluation previously outlined in the 2008 MRC publication: intervention implementation, causal mechanisms of impact and contextual factors [19, 27].

Process evaluation typically encompasses a combination of quantitative and qualitative methods, including structured observations, questionnaires, semi-structured interviews, focus groups, and logs [27]. Quantitative methods have the advantage of allowing for quick analysis and relatively straightforward interpretation; they can be useful in documenting the dose and reach of intervention activities [28]. However, these methods cannot be used for answering why or how an intervention component is delivered, and if it was delivered as initially planned. Qualitative methods have the advantage of allowing investigators to detail how activities are delivered–in context, elicit unanticipated information, suggest innovations that may improve intervention delivery, and to capture diverse perspectives of intervention stakeholders.

Researchers use systems science methods increasingly to examine process evaluation measures in schools [29]. These tools facilitate analysis of complex systems and processes, ones particularly useful for evaluating implementation of school-based interventions, as they can provide valuable information on how to optimise current and future implementation processes [30].

Many schools have limited and relatively fixed resources to undertake core tasks in addition to regular, and sometimes competing, demands for the introduction of new concepts, policies, programmes and activities [29]. Although the importance of process evaluation is widely recognised, researchers must avoid evaluations that excessively burden school staff, compromising intervention delivery [30].

In this study we provide a methodology for process evaluation of an Irish primary school-based PA and nutrition intervention. We examine the effectiveness of multicomponent public health interventions such as Project Spraoi that can vary depending on the specific intervention and the population it targets. As the purpose of this paper is to only report on the methods of process evaluation undertaken, overall outcomes of the Project Spraoi intervention are reported elsewhere [31].

## Data and methods

### The Project Spraoi description

Project Spraoi, an Irish, whole-school, multi-component primary school intervention aims to deliver 20 min extra daily PA to students during the school day and improve student’s nutritional knowledge and behaviours [7]. We assessed the primary outcome measures of the intervention (body mass index (BMI) standard deviation score (BMI z-score), waist circumference (cm), 550 m walk/run time (secs) and PA levels) only in children who at induction were in ‘senior infants’ (6–7 years of age) and ‘fourth class’ (9–10 years). The parent or guardian granted permission for each child to participate in the measurement element of the study. All children in the intervention schools participated in the intervention activities because the lead author delivered them to all students.

**Fig. 1:**
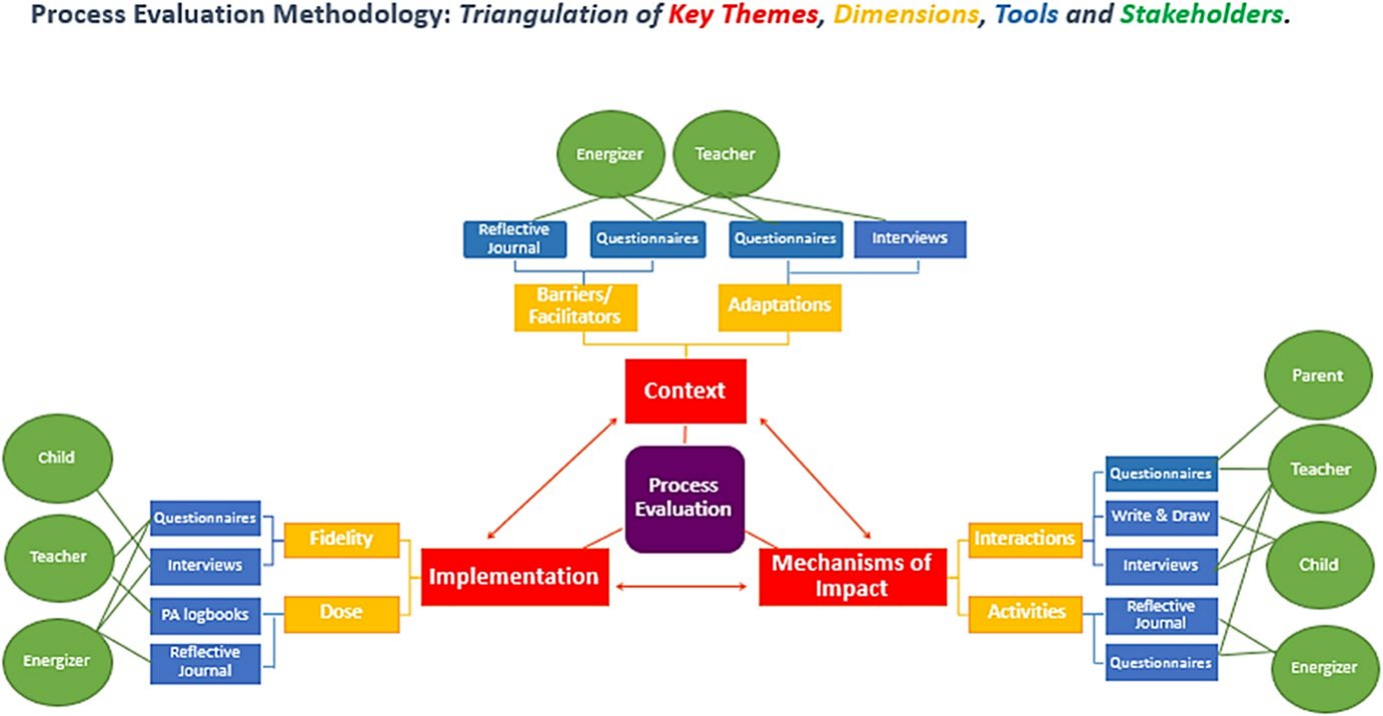
Process evaluation methodology of Project Spraoi

The intervention ran throughout the school year (September 2015 through June 2016) with the Energizer visiting the school for a maximum of 2 days per week. We developed an overview of the process evaluation methodology for Project Spraoi presented in Fig. 1.

We measured outcomes among the same senior infant and fourth class cohort of children at the beginning and end of the academic school year. Process evaluation ran concurrently to intervention delivery, with data collected at multiple timepoints throughout the school year (Table 1), using a variety of process evaluation data collection tools (Table 2).

**Table 1:**
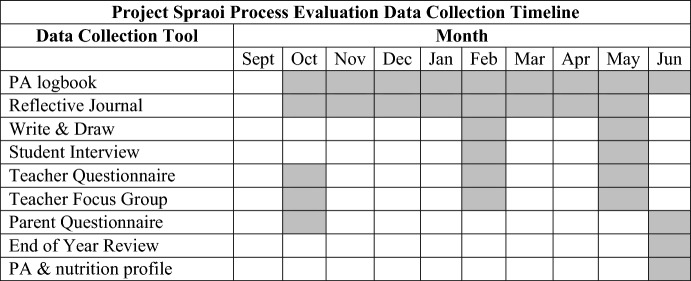
Process evaluation data collection timeline

**Table 2:** Process evaluation data collection tools

Measure	Evaluation dimension	Stakeholder	Methods	Source
Physical Activity Log	Dose delivered Barriers	Class teachers (intervention schools) (*n* = 63)	Teachers indicated by ticking a box the time spent (5, 10, 15 or 20 min) and type of activity (Huff and Puff, learning games, activity breaks or other) delivered to their class each day during a given week Teachers indicated which day and for how long physical education (PE) lessons were delivered each week by ticking the appropriate box and writing in the minutes of PE delivered If teachers did not deliver any activity on any given day, they indicated the reason why in a comments box	Griffin et al. (2014)
Write & Draw	Interactions Activities	Students (phase 2: all classes; phase 3: testing classes only) (*n* = 585)	Children completed a worksheet resembling a class activity that was titled ‘What Project Spraoi means to me’ Children drew a picture in the outlined drawing area Children then wrote on the lines below about what Project Spraoi means to them A ‘spelling holiday’ was granted for the duration of the task Reassurance was offered that this was not a test and drawings would not appear on display	McWhirter (2014)
Semi-structured interviews	Fidelity Interactions	Students (a sub sample of 2 students from each class that completed W&D) (*n* = 40)	Interviews were conducted 1 week after completion of the Write and Draw Questions were grouped under three headings; (i) write and draw, (ii) intervention activities and interactions with Project Spraoi, and (iii) the school environment Questions and interview protocol employed were influenced by the considerations outlined by Westcott et al. (2002; 2005). Open ended questions were favoured to encourage longer responses Interviews audio was recorded using a Dictaphone, transcribed verbatim and analysed using NVivo version 11 qualitative analysis software	Merriam (2001) McEvilly (2013)
Reflective Journal	Dose Activities Barriers Facilitators	Energizer (*n* = 5)	Section A included structured questions to quantify dose delivered and received by participants. Section A also documented what activities were delivered that week and how participants interacted with them using an enjoyment scale Section B used open ended questions that allowed Energizers the opportunity to openly and honestly reflect on their daily experiences as the interventionist (“What went well?/could be improved?”)	Griffin et al. (2014)
Questionnaires	Fidelity Interactions Activities Barriers Facilitators Adaptations	Teachers (Intervention & control schools) School Staff Energizer (*n* = 234)	Data collection involved the use of 5 different questionnaires: teacher questionnaire; PA & healthy eating profile; end of year review; energizer questionnaire and parent questionnaire Questions were presented as a set of statements with which respondents were asked to indicate their level of agreement using a 5-point Likert scale Open ended questions were used to gather information about stakeholders’ interactions with Project Spraoi, prompt any unanticipated information and suggest innovations to maximise intervention delivery and support at each site	Thomas et al. (2015)

### Evaluation process

**Fig. 2:**
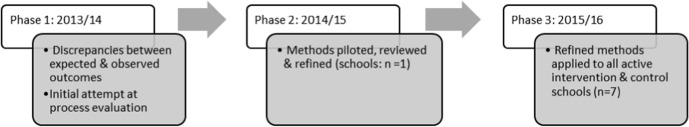
Progression of process evaluation of Project Spraoi

As the intervention evolved, so too did the scope of the process evaluation. We developed and refined methods for the process evaluation over three phases from 2013 to 2016 (Fig. 2).

The process evaluation of this study alone generated close to 2,000 data sheets from interviews (*n* = 40), PA logs (*n* = 630), reflective journals (*n* = 69), write & draw (*n* = 585) and questionnaires (391).

Following phase one of Project Spraoi (2013–2014), an impact evaluation of the intervention revealed discrepancies between expected and observed outcomes (unpublished observations). An initial attempt at process evaluation undertaken on a post hoc basis during the first phase did not yield sufficient data to allow for a valid explanation for the unexpected results observed ( substantial improvements in the control cohort) (unpublished observations by the Energizer/evaluator). Our inability to interpret phase 1 findings highlighted the need to implement and report a robust methodology to document the process by which the Project Spraoi intervention achieved the observed effects and to distinguish the relative contribution of each intervention component to overall outcomes, in context.

PENZ is a health promotion programme from New Zealand that sees a team of 26 ‘Energizers,’ working with their local schools and communities, to increase children's PA, improve nutrition, and enhance their overall health [8]. Due to the lack of any process evaluation data from PENZ, the authors, in consultation with the Project Spraoi research team, conducted a preliminary study during phase two, the second year of implementation (2014–2015) in site A, a rural, mixed gender school. We intended to inform the final design of the process evaluation methods and data collection tools. Following the phase two study, we expanded a refined robust methodology for process evaluation of Project Spraoi to include all intervention and control schools (*n* = 7) for the third year of implementation of Project Spraoi (2015/16) (phase three) (Table 3).

**Table 3:** Summary of data collected by questionnaires relative to process evaluation

Questionnaire	Stakeholder	Purpose	Evaluation Dimension					
			Fidelity	Dose	Adaptation	Barriers/ Facilitator	Inter- actions	Activities
Teacher Questionnaire	Class teachers (intervention) (*n* = 63)	Assess implementers self-perceived delivery of each intervention component Identify contextual barriers/facilitators Suggest innovations	✓			✓	✓	✓
Energizer Questionnaire	Active Energizers (*n* = 5)		✓	✓	✓	✓	✓	✓
Physical activity & nutrition profile	Evaluation class teachers (control & intervention) (*n* = 16)	Understand school context and activities undertaken by school outside of PS which may influence outcomes Compare control context to the intervention context				✓		✓
End of year review	All school staff (intervention) (*n* = 85)	Note changes to the school environment incl. student behaviour & staff morale			✓	✓	✓	

Before beginning the process evaluation of Project Spraoi, the research team needed first to define the intervention, its activities and theory of change and distinguish both how we expected the effects of each specific intervention activity to occur and how these effects might be replicated by similar future interventions [24]. As already stated, Project Spraoi is based on a proven methodology, PENZ, and as such, the theories of change upon which Project Spraoi is based were set out by its NZ counterpart.

The process evaluation of Project Spraoi employed the three themes described by the British MRC guidance for process evaluation: context, implementation and mechanisms of impact [19, 23]. These themes are described in more detail in Supplementary Material Part 1. Each theme was further subcategorised into three key dimensions for evaluation by the authors: reach, fidelity and dose, which are explained in more detail below.

The HEALTHY study [25] was a large-scale multicomponent school-based intervention targeting both diet and PA using a combination of observations of intervention sessions, interviews and focus groups (with school staff and children), alongside teacher feedback forms on class behaviour, in their evaluation. Such a diversity of methods allowed for triangulation of data from different sources and as a result, the authors of Project Spraoi chose to follow a similar approach. As a result, in line with the HEALTHY study, the process evaluation of Project Spraoi opted to omit the analysis of ‘reach’ [25]. ‘Reach’ evaluates how widely the intervention is adopted within the intended population. This dimension was fixed throughout the course of the intervention, due to the programme being delivered under controlled conditions to all classes in intervention schools each year; that is, students did not have the opportunity to ‘opt out’ of the intervention, as the project applied to all students in the school. Therefore, implementation of Project Spraoi was analysed by the authors using two (fidelity and dose) of the three (fidelity, dose, reach) dimensions described by Linnan & Steckler [26].. Fidelity refers to the degree to which an intervention is implemented as planned or intended. It ensures that the programme is delivered consistently, aligning with its original design and objectives [27]. Dose refers to the amount of an intervention delivered or received during an intervention [27].

We categorized adaptations (intentional modifications made to the Project Spraoi intervention when it was delivered) under three headings, ‘innovation’, ‘drift’ and ‘subversion’ [27] in line with the FRAME structure [28], which supports research on the timing, nature, goals, and impact of adaptations to evidence-based interventions. In the Project Spraoi context, innovation relates to the implementation of the PA and healthy eating components that intended to lead to positive change or improvement. Drift refers to any unintended deviations that took place in the school delivery which were different from the intended design of the intervention. Finally, subversion refers to any deliberate efforts to undermine or alter the intended purpose of an intervention or policy. It may involve resistance, intentional modifications, or circumvention of established procedures.

Fidelity (whether Project Spraoi was delivered as planned) was evaluated by the authors using questionnaires and interviews with intervention implementers (Energizers and teachers) and participants (students) (see Table 2). Interviews were conducted 1 week after completion of the Write and Draw task for students and questions were grouped under three headings; (i) write and draw, (ii) intervention activities and interactions with Project Spraoi, and (iii) the school environment. The questionnaire questions were presented as a set of statements with which respondents were asked to indicate their level of agreement using a 5-point Likert scale. Open ended questions were used to gather information about their interactions with Project Spraoi, prompt any unanticipated information and suggest innovation/s to maximise intervention delivery and support at each site.

Dose was evaluated by the authors using PA logs and an Energizer reflective journal to quantify the total minutes of extra daily PA and number of healthy eating lessons delivered by implementers of the Project Spraoi intervention. Teachers indicated, by ticking a box, the time spent (5, 10, 15 or 20 min) and type of activity (Huff and Puff, learning games, activity breaks or other) delivered to their class each day during a given week. Teachers also indicated which day, and for how long, physical education (PE) lessons were delivered each week by ticking the appropriate box and writing in the minutes of PE delivered. If teachers did not deliver any activity on any given day, they indicated the reason why in a comments box.

The Energizer reflective journal included structured questions to quantify dose delivered and received by participants. It also documented what activities were delivered that week and how participants interacted with them using an enjoyment scale. Open ended questions that allowed Energizers the opportunity to openly and honestly reflect on their daily experiences as the interventionist (“What went well?/could be improved?”) were also included.

To evaluate the influence of context on the delivery of Project Spraoi we focused on two evaluation dimensions, (i) barriers (obstacles or challenges that hindered the successful implementation of the project) and facilitators (factors that enhanced or supported the implementation of Project Spraoi) and (ii) adaptations (intentional modifications made to the intervention). Questionnaires and focus groups with implementers of Project Spraoi, Energizers and teachers, were conducted by the lead author and analysed throughout the course of the intervention year (phase 3) to document and track adaptations made to implementation of the intervention across sites. This information enabled Energizers to make mid-course adaptations to intervention delivery in response to individual teacher’s perceived barriers and facilitators. These adaptations were then documented by the lead author using Energizer’s reflective journals and questionnaires.

## Results

Overall, intervention fidelity was low, as teachers delivered, on average, 50–80% of the prescribed daily PA. There was considerable variability in how Project Spraoi teachers delivered the intervention and students received it, but adaptations that were made to it during its delivery facilitated intervention delivery.

PA logs were completed weekly by all class teachers (*n* = 11) during year 2. Teachers indicated by ticking the box, the type and time, either 5, 10, 15 or 20 min of activity delivered by them each day. The mean amount of extra daily PA (mins) delivered by teachers during year two of Project Spraoi was analysed using a between and within subject’s ANOVA test. The within subjects’ factor was time and the between subjects’ factor was the class taught by the relevant teacher. Teachers who taught class groups from junior infants to second class were assigned to the junior category (*n* = 6), while teachers who taught class groups from third class to sixth class were assigned to the senior category (*n* = 5). Over the course of the academic year, the mean amount of extra daily PA delivered by teachers each month varied significantly (*p* < 0.05, *n*^2^ = 0.68). The interaction effect of time and class group taught indicated that over the course of the school year, junior class teachers delivered significantly more extra daily PA than teachers of senior classes (*p* = 0.002, *n*^2^ = 0.355).

A Bonferroni post hoc test was conducted to explore the monthly difference in the amount of extra daily PA delivered by teachers within the eight months of intervention implemented in year two. Although the increase in the amount of extra daily PA delivered by teachers over time did not occur in a linear manner, a statistically significant mean increase of 10.9 min (*p* = 0.005) was reported between November and June. The largest increase in the amount of extra daily PA delivered by teachers was between April and June (mean difference = 11.5 min, *p* = 0.021).

Further analysis of the PA logs using a one-sample t-test revealed discrepancies in the fidelity of intervention delivery by class teachers. The mean amount of extra PA delivered by teachers daily (12.2 min) was significantly lower than the PS target of 20 min from November to May (*p* < 0.05). Both groups did not achieve the target amount of extra daily PA until June.

**Fig. 3:**
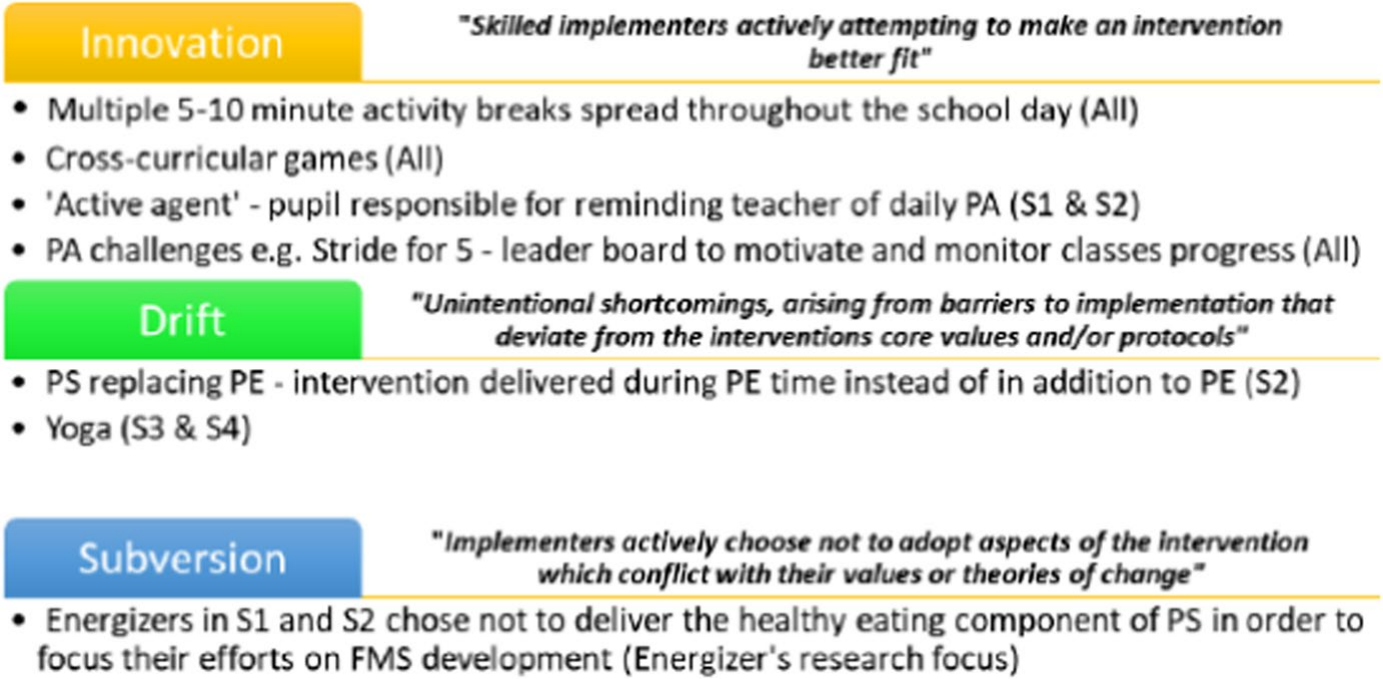
Summary of facilitator level (Energizer) adaptations to intervention delivery

Adaptations to the PS intervention as recorded in the Energizers’ reflective journals (*n* = 5) were categorised as either innovation, drift or subversion. These adaptations, which are summarised in Fig. 3, were implemented in response to teacher feedback.

## Discussion

We described the process evaluation methods used in Project Spraoi, which combined methods and implemented guidance from the literature. Although process evaluation is in its nature complex, the authors believe that the approach developed as part of this study (Fig. 1) provides an easy framework for future interventions to map their process evaluation methods according to the guidance set out by the British MRC [19, 23].

Through the examination of procedures linked to each individual intervention component, the authors can now identify which components dominated the effects observed. Knowledge gained from the wider process evaluation, specifically the qualitative examination of contextual influences on intervention delivery and response, allowed us to assess the impact of context on outcomes. This coincides with the additional benefit the data provided on informing how best to implement the intervention more widely, beyond the study setting.

### Limitations

There are limitations to the methods described and we encountered several barriers to data collection. It was not possible to fully assess the validity and reliability of the logbooks, reflective journals, and questionnaires as no gold standard exists for fidelity assessment; the nature of process evaluation means many tools are intervention specific. To overcome this barrier, as previously described, during phase two (2014/15), tools we rigorously piloted and refined in response to feedback and we also conducted inter-rater reliability tests.

In general, comprehensiveness of process evaluation poses a major challenge due to the amount and range of data collected. There is a huge quantity and many types of data, each requiring specific attention during data cleaning and analysis. We recommend that future studies provide electronic versions of the data collection tools, such as the PA logs and questionnaires, as this would make later analyses more efficient. We also recommend that researchers plot out the analysis of data collected as part of the overall methods for evaluation [19]. A time-benefit analysis would also be useful during the piloting phase, particularly if resources and researcher time is limited.

Similar to the WAVES study [32], a large-scale multicomponent school-based intervention targeting both diet and PA, a major challenge and limitation to process evaluation methodology was identified. The additional workload that the process evaluation created for teachers and Energizers who were delivering the intervention, particularly in relation to the logbooks and reflective journal [14], was reported. As a result, these tools were refined by the lead author to employ a ‘tick the box’ style, which minimised completion time. An electronic version of the PA log was also given to the teachers, upon request, as some found the additional paper burdensome. Even after taking these measures to try to optimise data collection, completion of the logs and reflective journals remained erratic by the teachers and Energizers and as a result, their data were incomplete in parts. A significant difference (*p* < 0.05) in mean completion rates was also noted by the lead author between intervention schools, which at times, made comparisons between sites difficult.

Time constraints and access restrictions also posed a barrier when trying to undertake qualitative analyses with staff. Where possible, qualitative data collection (focus groups) were completed by the lead researcher with all available staff together, during lunch hours or immediately after school. At one school, policy did not permit any lunch time or post school meetings with research staff, thus teachers completed questionnaires one on one with the researcher during each class’ designated Spraoi time that week. To maintain the integrity of the researchers’ relationship with the schools and prioritise intervention delivery, the author/s conducted these qualitative analyses at a time and place convenient to school personnel. For future research, the authors advise that investigators plan qualitative data collection in advance with school staff, taking into consideration school events, exams, and class excursions.

### Strengths

Data were collected by the lead author for each evaluation dimension using multiple tools from a variety of sources. This enabled triangulation of findings, which previous studies have been found to improve the trustworthiness of qualitative data and to help avoid potential bias in reporting [33]. If data from two different sources provided conflicting information, a judgement was made by the lead author regarding which source was more reliable.

The multi-pronged process evaluation design provides a comprehensive overview of the Project Spraoi intervention and how participants delivered it. The rigorous piloting and refining of methods and data collection tools continuously during phase two (2014–2015) was a key asset of this study. This resulted in robust and efficient final methods for all schools (*n* = 5) in phase three (2015–2016) because they allowed us to maximise data collection and to minimise workload for staff. There is no easy way to analyse a multi-component intervention, as it is complex in nature and thus requires a complex evaluation [19, 23]. However, we believe that the developed template devised for this study provides a workable mapping tool for process evaluation, which may be transferable to future studies.

Recent studies have suggested that process evaluations of school-based trials provide information relating to the control cohort, which may influence outcomes [34]. The PA and Nutrition Profile questionnaire completed by both the control and intervention evaluation class teachers provided this study with valuable information about the control school and allowed the researchers to make informed comparisons between the intervention and control context, thus strengthening the interpretation of the overall trial outcomes.

Although we noted an increase in the number of process evaluation studies reported in the literature in recent years, Ireland still lags. This study is the first in Ireland to provide a comprehensive process evaluation of a multi-component school-based health promotion intervention. The findings of this study call for future exploration of artificial intelligence (AI) and machine learning (ML) tools to facilitate study evaluation and the developed framework for further methodology improvements.

## Conclusion

This paper reports a detailed methodology for process evaluation of a complex school-based health promotion intervention. It builds upon the current literature and provides researchers with a workable framework for future process evaluations in the primary school setting.

## Supplementary Information

Below is the link to the electronic supplementary material.

Supplementary file1 (DOCX 113 kb)

## Data Availability

The datasets used and/or analysed during the current study are available from the corresponding author on reasonable request.
