# Supplementary material for: Methodology for evaluation of complex school-based health promotion interventions
Source: J Public Health Policy. 2024 Aug 12;45(4):623–38. doi: 10.1057/s41271-024-00510-4 (PMC11609087; doi:10.1057/s41271-024-00510-4)
Supplement: Supplementary file 1 — Supplementary file1 (DOCX 113 kb) [file 41271_2024_510_MOESM1_ESM.docx]

*Original article*

**Methodology for Evaluation of Complex School-Based Health Promotion Interventions**

Yvonne O’Byrne^a^, Joan Dinneen^a^ , and Tara Coppinger^a^*

^a^Sport, Leisure & Childhood Studies, Munster Technological University, Cork, Ireland

**Supplementary Material**

**Part 1.**

The MRC guidance for process evaluation of complex interventions [1] was essential for the Project Spraoi research team to understand how the intervention worked, the school context in which it operated and its mechanisms. The Framework focuses on three themes: (i) Implementation - how well the intervention was delivered; (ii) Context: the environment in which the intervention occurs and (iii) Mechanisms of Impact - how the intervention produces change. Figure 4 outlines these themes in more detail and in order for the team to investigate these components effectively, our clear intervention description was followed, which then informed the interpretation of the intervention outcomes.


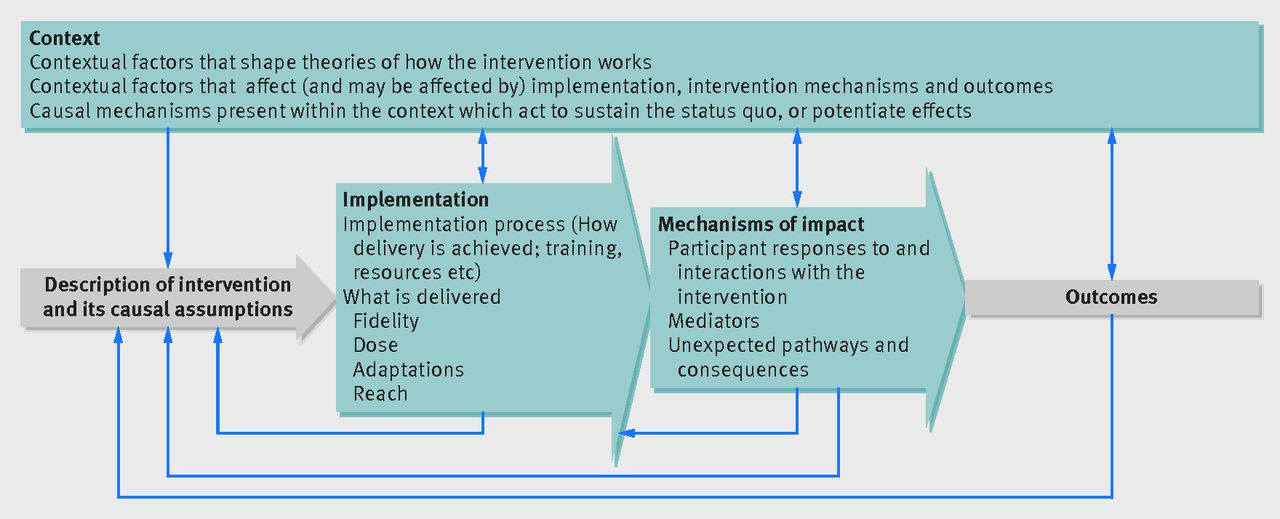


Figure 4: Key functions of process evaluation and relations among them (Moore et al. 2015)

1. *Implementation*

In order to determine and conclude what has worked in an intervention, process evaluations often try to capture ‘fidelity’, which is whether the intended delivery of the intervention has been achieved [1, 2]. Checking for fidelity ensured that the Project Spraoi intervention was delivered consistently, aligning with its original design and objectives. The research team also examined ‘dose’ (the amount of the intervention that was delivered) in order to understand the impact of the programme’s health promotion efforts. as well as their ‘reach’ [2, 3].

Carroll et al. [4] outlines fidelity as a mixture of frequency, content, length of delivery, as well as coverage. Though using different terminology, this definition is similar to the definition of ‘implementation’ by Linnan and Steckler [3]; recognised as a combination of fidelity, dose and reach. Yet, the Carroll et al. [4] framework delves deeper by inviting researchers to examine the processes such as how effectively resources are applied. Consequently, this framework highlights the need to consider the complexity of an intervention, its comprehensive description, how effective the strategies are to support its implementation, as well as the participants response to the intervention when undertaking evaluations.

Currently, there is no consensus on how best to divide the study of implementation into key subcomponents (such as fidelity, dose, and reach) [1]. This may be due to the many combinations of varying subcomponents and different terminology included in the evaluation of implementation across the different process evaluation frameworks and the wide range of differing process evaluation methods reported in trials [5].

1. *Context*

The context component in process evaluation is essential in order to understand why and how an intervention failed or succeeded. It is usually assessed by determining which environmental factors affect the program implementation, Yet, to date, context has not been sufficiently explored and reported [6]. Context is measured in RE-AIM when reporting on Reach. To assess Reach, the researcher must also report on the target population, which necessitates a description of the eligible study population and its context. Measuring reach can help identify whether the intervention is being implemented as intended, whether it is being delivered to the intended population and whether it can be replicated in other settings and populations [7].

Understanding the role of context in process evaluation is crucial as environmental factors may influence intervention implementation or outcomes [8]. Indeed, interventions may vary in different contexts to adjust to barriers or facilitators posed by different external environments. Furthermore, interventions implemented in exactly the same way in separate contexts may also produce different results due to contextual influences [9]. This may be due to the differing sources of problems targeted by interventions.

Complex interventions work by introducing mechanisms that are adequately suited to their context to produce change [10]. Often intervention implementation may need to be tailored to achieve better contextual fit [1]. However, adaptations made to the implementation of intervention components in new contexts must be examined to ensure that they align with the theory of the intervention and do not undermine intervention fidelity [11].

1. *Causal Mechanisms of Impact*

Causal mechanisms of impact may simply be described as the intervention’s theories of change [1]. Complex interventions usually have multiple related intervention components which may each contribute individually or collectively to different aspects of the overall trial outcomes. Exploring the mechanisms through which interventions bring about change is crucial to understanding both how the effects of each specific intervention component occurred and how these effects might be replicated by similar future interventions [7]. The 2008 MRC guidance for developing and evaluating complex interventions argues that only through scrutiny of causal mechanisms is it possible to develop more effective interventions, and to understand how findings might be transferred across settings and populations [1].

Rather than passively receiving interventions, participants interact with them, and outcomes are produced by these interactions in context [10]. Therefore, understanding how participants interact with complex interventions is crucial to understanding the mechanisms through which the intervention works [1].

**Part 2.**

#### (a) Write and draw

Write and draw is an inclusive tool for classroom-based research in health education, enabling even the youngest children to participate and contribute their experiences and unique perspectives [12, 13]. It’s resemblance to a classroom activity makes it most useful in a primary school setting.

Write and draw was delivered to all students in testing classes (junior & senior evaluation cohorts) in intervention schools by the researcher. In addition to their original consent to participate in Project Spraoi, further consent was sought on three levels; school, teacher and child. Teachers were asked to sign a consent form for their class to participate. Children were given the opportunity to consent to participate on their own behalf by simply raising their hand. To avoid children feeling coerced into participation, a separate task of colouring or drawing on a blank sheet was offered as a substitute activity. This task was chosen as it would neither be a reward nor a punishment for opting out of participation in the write and draw evaluation [13].

Possible limitations of using drawings as a source of feedback are that they only represent experiences and views that can be represented graphically and are limited by the individual’s skills [13]. To avoid this, drawings were used in conjunction with interviews. Thus, children were not limited to expressing experiences that could only be represented graphically, and they had the opportunity to explain their pictures, avoiding any interpretation issues. Indeed, it did not matter if drawings were unclear, as similar to McEvilly [14], drawings were used to encourage conversation and generate interview data, rather than as a source of data in themselves. The writing which accompanied the drawing was included in qualitative analysis to assess student’s interactions with the Project Spraoi intervention. This data was evaluated using computer assisted qualitative analysis software, Quirkos (version 2).

#### (b) Interviews

Despite much qualitative research involving the use of purposive sampling, random sampling was selected for this component to negate charges of researcher bias in the selection of participants [15, 16]. Two children from each class, one girl and one boy if the school was mixed, were selected to participate. The interviews were led by a member of the Project Spraoi research team, accompanied by a research assistant. In addition to their original consent, further consent was sought by both parent/guardian and child to participate in the interview and have audio recorded.

Interviews were conducted in a private quiet space, often a teacher resource room, separate to the main classroom. For ethical reasons, two children were present in the interview room at all times with two members of the Project Spraoi research team (the interviewer and a research assistant). One child was questioned at a time by the interviewer, while the other participated in a colouring activity with the research assistant. The participant was presented with their completed write and draw work sheet and asked to talk about their drawing. This protocol was adopted to break the perceived power imbalance between interviewer and interviewee and generate open conversation about Project Spraoi [14]. Questions were trialled during the preliminary study (May 2015) and refined/rephrased for clarity as needed. Each interview lasted on average 10minutes 15 seconds (±2.38mins).

#### (c) Questionnaires

Similar to Griffin et al., [17], multiple questionnaires were developed for completion by school staff, and Energizers throughout the course of the academic year. Following recommendations from the literature [17], questionnaires were condensed to a single A4 size page, using ‘tick the box’ style questions to maximise response while minimising burden and additional workload for school staff. As questionnaires were specific to the implementation of Project Spraoi, questions were piloted in the preliminary study (2014/15) with teachers in school A (n=11) before being used across all intervention schools the following year (2015/16). A one-week test-retest evaluation was conducted during the pilot phase with a sub-sample of teachers from school A (n=7) to assess the repeatability and reliability of the Likert scale ‘attitude’ questions. The reliability coefficient for teacher surveys ranged between .78 and .91.

**References**

1. Moore G, Audrey S, Barker M, Bond L, Bonnell C, Hardeman C, Moore L, O’Cathain A, Tinati, T, Wight D, Baird J. Process evaluation of complex interventions: UK Medical Research Council (MRC) guidance. BMC. 2015; 350.
2. Patton, M.Q., Developmental Evaluation: Applying Complexity Concepts to Enhance Innovation and Use, 2011, Guilford Press: New York, NY, USA.
3. Linnan LE, Steckler AE. Process evaluation for public health interventions and research. San Francisco: Jossey-Bass; 2002.
4. Carroll, C., Patterson, M., Wood, S., Booth, A., Rick, J. and Balain, S. A conceptual framework for implementation fidelity, IS, 2007, 2, 1, 40.
5. Glasgow RE, Vogt TM, Boles SM. Evaluating the public health impact of health promotion interventions: the RE-AIM Framework, Am J Public Health, 1999; 9:89.
6. Durlak, J.A. and Dupre, E.P. Implementation matters: A review of research on the influence of implementation on program outcomes and the factors affecting implementation, AJCP, 2008, 41, 3-4, 327-350.
7. Grant, A., Treweek, S., Dreischulte, T., Foy, R., Guthrie, B. Process evaluations for cluster-randomised trials of complex interventions: a proposed framework for design and reporting, Trials, 2013, 14, 15.
8. Saunders, R., Evans, M. and Joshi, P. Developing a process-evaluation plan for assessing health promotion program implementation: A how-to guide, HPP, 2005, 6, 134-147.
9. Shiell A, Hawe P, Gold L. Complex interventions or complex systems? Implications for health economic evaluation. BMJ. 2008, 7;336(7656):1281-3.
10. Pawson, R. and Tilley, N. Realistic evaluation, 1997, London: Sage.
11. Hawe P, Shiell A, Riley T. Complex interventions: how ‘out of control’ can a randomised controlled trial be?” BMJ, 2004; 328: 7455.
12. McWhirter J. The draw and write technique as a versatile tool for researching children's understanding of health and well-being, IJHPE. 2014; 5:52.
13. MacPhail A, Kinchin G. The use of drawings as an evaluative tool: students’ experiences of sport education, Phys Educ Sport Peda. 2004; 1:9.
14. McEvilly N. Investigating the place and meaning of Physical Education to preschool children: methodological lessons from a research study”, Spor Educ Soc, 2013; 3: 20.
15. Preece R. Starting research: an introduction to academic research and dissertation writing, London: Pinter; 1994.
16. Shenton AK. Strategies for ensuring trustworthiness in qualitative research projects, Educ Inf. 2004; 22(2):63–75.
17. Griffin, T.L., Pallan, M.J., Clarke, J.L., Lancashire, E.R., Lyon, A., Parry, J.M., Adab, P. Process evaluation design in a cluster randomised controlled childhood obesity prevention trial: the WAVES study, IJBNPA, 2014, 11, 112.
